# Precise Anatomic Localization of Accumulated Lipids in ***Mfp2*** Deficient Murine Brains Through Automated Registration of SIMS Images to the Allen Brain Atlas

**DOI:** 10.1007/s13361-015-1146-6

**Published:** 2015-04-28

**Authors:** Karolina Škrášková, Artem Khmelinskii, Walid M. Abdelmoula, Stephanie De Munter, Myriam Baes, Liam McDonnell, Jouke Dijkstra, Ron M. A. Heeren

**Affiliations:** FOM-Institute AMOLF, Amsterdam, The Netherlands; TI-COAST, Amsterdam, The Netherlands; Percuros B.V., Enschede, The Netherlands; Division of Image Processing, Department of Radiology, LUMC, Leiden, The Netherlands; Laboratory of Cellular Metabolism, KU Leuven, Leuven, Belgium; Center for Proteomics and Metabolomics, Leiden University Medical Center, Leiden, The Netherlands; Fondazione Pisana per la Scienza ONLUS, Pisa, Italy; M4I, The Maastricht MultiModal Molecular Imaging Institute, University of Maastricht, Maastricht, The Netherlands

**Keywords:** SIMS mass spectrometry imaging, Allen Brain Atlas, Image registration, Lipid accumulation

## Abstract

Mass spectrometry imaging (MSI) is a powerful tool for the molecular characterization of specific tissue regions. Histochemical staining provides anatomic information complementary to MSI data. The combination of both modalities has been proven to be beneficial. However, direct comparison of histology based and mass spectrometry-based molecular images can become problematic because of potential tissue damages or changes caused by different sample preparation. Curated atlases such as the Allen Brain Atlas (ABA) offer a collection of highly detailed and standardized anatomic information. Direct comparison of MSI brain data to the ABA allows for conclusions to be drawn on precise anatomic localization of the molecular signal. Here we applied secondary ion mass spectrometry imaging at high spatial resolution to study brains of knock-out mouse models with impaired peroxisomal β-oxidation. Murine models were lacking D-multifunctional protein (MFP2), which is involved in degradation of very long chain fatty acids. SIMS imaging revealed deposits of fatty acids within distinct brain regions. Manual comparison of the MSI data with the histologic stains did not allow for an unequivocal anatomic identification of the fatty acids rich regions. We further employed an automated pipeline for co-registration of the SIMS data to the ABA. The registration enabled precise anatomic annotation of the brain structures with the revealed lipid deposits. The precise anatomic localization allowed for a deeper insight into the pathology of *Mfp2* deficient mouse models.

**Electronic supplementary material:**

The online version of this article (doi:10.1007/s13361-015-1146-6) contains supplementary material, which is available to authorized users.

**Graphical Abstract:**
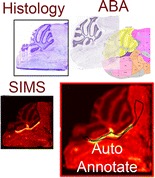
ᅟ

## Introduction

Molecular characterization and classification of specific tissue regions has become crucial in understanding of the mechanisms of various tissue pathologies. In the past decade, mass spectrometry imaging (MSI) has proven to be a tremendously useful technique for the spatial investigation of molecular tissue patterns [1–5]. The uniqueness and strength of MSI lie in the ability to simultaneously localize and identify hundreds of molecules from a tissue surface in a single experiment. MSI is also referred to as molecular histology [2, 6]. It can tell apart distinct tissue regions even if they are not differentiated by any of the established histochemical methods. MSI allows for partitioning of the tissue surface into regions with the same or similar mass spectrometric (MS) profiles. Hence, it enables the investigation of molecular changes that occur prior to or without any morphologic changes [7] and the identification of hidden anatomic features.

The better the spatial resolution of a MSI experiment, the finer structures of the tissue sample can be revealed. Currently, the best spatial resolution within the MSI field is provided by secondary ion mass spectrometry (SIMS) instruments. The spatial resolution achievable with SIMS reaches well below 1 μm [4]. SIMS makes use of a primary ion beam to probe the sample surface. The mass range of the examined molecules is limited because the impact of the atomic ion beam with the substrate causes extensive molecular fragmentation. Modern molecular ion or cluster ion beams limit the extent of fragmentation. SIMS has been used for the investigation of lipids and inorganic fragments in the study of several biological samples [2, 4].

Employment of multivariate statistical analysis to MSI datasets helps to reveal/highlight MS-profiled tissue regions. Principal component analysis (PCA) and clustering methods are frequently used in probing the enormous MSI data load [3, 8]. Results of such a computational segmentation are commonly compared with the results obtained by (immuno)histochemical staining of the same or an adjacent tissue section. The complementary information provided by MSI and histology can then be advantageously combined. MS-acquired and the histology-based optical images are typically co-registered for an accurate and efficient comparison. So far this has mostly been done manually with the guidance of fiducial markers or using clear anatomic detail [9, 10]. Matusch et al. took advantage of a commercially available software (Pmod) for alignment of histology and MS images [11]. The general unavailability of the program, however, prevents its common use in academia. Recently, progress was made by automating the histology-MSI co-registration process. Abdelmoula et al. [12] described methodology based on t-distributed stochastic neighbor embedding (tSNE) technique. tSNE is a nonlinear dimensionality reduction technique developed by van der Maaten et al. [13]. It highlights specific anatomic regions representing the global MSI dataset. The regions can be subsequently used as markers for the alignment. It was demonstrated [12] that tSNE representation of MSI data reveals enough anatomic details that can be treated as landmarks for its registration to histology.

Various folds, tears, or general surface changes of the tissue sections, resulting from sample preparation or from MSI measurements, can significantly hamper their histologic evaluation. This phenomenon is accentuated when dealing with fine structural details. It becomes thus plausible to compare MSI datasets to curated anatomic atlases. These contain highly standardized data that describe the anatomy of particular tissue types in high detail. Rodent brain remains one of the most frequently employed tissue types analyzed with MSI. Rodents are often used as models of various neurologic and neurodegenerative diseases [2], and also serve as a model organism for mammalian brain development studies and behavioral genetics [14]. An example of such a curated atlas commonly employed in brain research is the Allen Brain Atlas (ABA) (http://www.brain-map.org/) [14]. ABA is a freely accessible database of gene expression and neuroanatomic reference data in mouse brain, developing mouse brain, and human brain [10]. ABA provides standardized high-resolution histologic images combined with hierarchically organized taxonomy of the respective brain regions. Combination of the histologic and anatomic information extracted from the ABA with the molecular information provided by MSI can become a powerful tool for brain tissue analysis, and in chemical neuroscience. A couple of papers recently published describe pipelines for alignment of MSI and the ABA data [10, 15]. The methodologies are based on the use of a histochemically stained tissue section that was previously MS imaged. The stained sample serves as an intermediate between the ABA and MSI data. Once MS and ABA images are aligned, direct conclusions about anatomic localization of MS signal and the affected genetic pathways can be made.

Here, we show how a combination of MSI and ABA data enables researchers to gain insights into the pathology of knock-out (KO) mouse models with impaired peroxisomal β-oxidation. In our experiments, *Mfp2* (multifunctional protein 2) deficient mouse models were involved. MFP2 is a protein that is responsible for formation of bile acids and the degradation of pristanic acid and the very-long chain fatty acids (VLCFA) [16]. *Mfp2*^-/-^ mice suffer from severe neuromotor dysfunctions and die before the age of 6 mo [17]. Histologically, severe neuroinflammation and cerebellar degeneration are observed. However, it remains obscure which peroxisome-dependent metabolite is responsible for the pathologies. Analysis of total brain lipid extracts of *Mfp2*^-/-^ mice revealed increases in the C26:0 content of phospholipids and in minor ceramide and sphingomyelin species. Also in the cerebellar phospholipids, C26:0 was increased, and histochemistry showed the presence of lipid droplets, especially within ependymal cells along the entire ventricular system and in the molecular layer of the cerebellum. In order to obtain spatial information about molecular abnormalities (either accumulation or deficits) brain sections of three biological replicates were MS-imaged at high spatial resolution using SIMS. PCA revealed accumulation of fatty acids (FA) in a sharply defined region underneath the cerebellum. Since manual comparison of the SIMS data with the histologic staining did not allow for an unequivocal identification of the brain region with the lipid deposits, the MS images were further co-registered to the ABA. The fully automated pipeline of the co-registration enabled precise anatomic annotation of the FA hotspot. This finding offered deeper insights into the pathologic mechanism of the *Mfp2* deficiency.

## Methods

### Mouse Breeding

The generation and breeding of *Mfp2*^*-/-*^ mice have been described in detail elsewhere [17]. Mice were bred in the specific-pathogen free animal housing facility of KU Leuven, had ad libitum access to water and standard rodent food, and were kept on a 12 h light and dark cycle. All animal experiments were performed in accordance with the “Guidelines for Care and Use of Experimental Animals” and fully approved by the Research Ethical Committee of the KU Leuven (177/2012).

### Tissue Preparation

Twelve-week old *Mfp2*^*-/-*^ mice and a wild type (WT) littermate were euthanized by an intraperitoneal Nembutal injection. Mice were perfused with cold ammonium acetate (50 mM, pH 7.3) in a 4°C room to prevent lipid degradation. Brains were rapidly removed, cut in the midsagittal plane, and frozen on dry ice. Brains were kept in glass vials at –80°C before further processing. Contamination and lipid diffusion were avoided by washing the working space and instruments with hexane.

### Tissue Preparation for SIMS-MSI

Tissue sections, 12-μm thick, were obtained using cryomicrotome (Microm International, Walldorf, Germany). The sections were thaw mounted [18] onto indium tin oxide (ITO) covered glass slides (Delta Technologies, Loveland, CO, USA). The tissue sections were stored at -80°C until further analysis. Prior to MSI, the tissue sections were placed into a vacuum desiccator for 20 min to reach room temperature while avoiding condensation of water on top of their surface. The tissue sections were covered with a 1 nm gold layer using a Quorum Technologies SC76440 sputter coater (New Haven, UK). Gold coating enhances the molecular signal in metal-assisted SIMS through increases in stopping power of the primary ions and an improvement of the surface conductivity.

### SIMS Mass Spectrometry Imaging

The samples were analyzed on TRIFT II (Physical Electronics, Chanhassan, MN, USA) time of flight secondary ion mass spectrometer (TOF-SIMS) equipped with an Au liquid ion metal gun, producing a 22 keV Au^+^ primary ion beam. A region of interest (roughly 6 × 6 mm) that included the cerebellum and brain stem was imaged in a mosaic mode consisting of 64 × 64 square tiles. Each tile contained 256 × 256 pixels. The size of a tile was 98.2 μm, 106.3 μm, and 82.8 μm, for the three knock-out replicates (KO1, KO2, KO3), respectively. The size of a tile for the WT mouse was 78.1 μm. The spatial resolution of the raw data was well below 0.5 μm. Acquisition time per tile was set to 11.4 s. Spectra were acquired in negative mode within the mass range of 1–1,000 Da. Spectra were first calibrated on low mass fragments (O, Cl, Au), and then recalibrated on gold clusters.

### Data Pre-Processing

The data was converted into MATLAB format employing our in-house developed ChemomeTricks toolbox for MATLAB (MathWorks, Natick, MA, USA). Mass channels were binned into 0.05 Da wide mass bins. An average spectrum of all pixels was used for subsequent peak picking. Peak picking was performed on a base peak mass spectrum with an algorithm, which is described in detail elsewhere [19]. The peak list contained 879, 1065, and 1443 peaks for KO1, 2 and 3, respectively, and 1400 peaks for the WT. The peak lists were used to integrate each pixel’s mass spectrum within the respective raw datasets. During the integration, the pixels were spatially binned resulting in 256 × 256 pixels datasets. A multiorder correction algorithm based on linear discriminant analysis was applied to remove image distortions caused by the mosaic character of the data acquisition. The algorithm is described in detail elsewhere [20]. The number of iterations differs according to the data quality. The number of iterations for presented datasets was 3, 7, and 8 for the three KO replicates, respectively, and 5 for the WT. Finally the SIMS data was recalibrated on gold coating related peaks with well-known *m/z* values in MATLAB environment using polynomial fit.

### Principal Component Analysis and Hierarchical Cluster Analysis

The pre-processed data was further used for PCA and hierarchical cluster analysis (HCA), which were both performed using the ChemomeTricks toolbox. The data was normalized on TIC and autoscaled prior to PCA. (*N.B.* By autoscaling we refer to a process in which each column of the data matrix was divided by its standard deviation and its mean was subtracted.) The first principal component separating the tissue from the surrounding was used to remove pixels of the latter. HCA was performed with the first 20 PCs employing the correlation distance and the average linkage approach.

### Tissue Staining

After SIMS imaging the tissue was stained with Cresyl violet (Nissl stain) and scanned using a Mirax DESK digital slide scanner (Zeiss, Germany).

### MSI data registration to the Allen Brain Atlas

The pipeline used to automatically register the SIMS datasets to the ABA is based on a combination of two recently published techniques developed by Abdelmoula *et al.*: 1.) automatic registration of MSI data to histology using tSNE [12] and 2.) the automatic registration of histology to the ABA [10]. The whole co-registration pipeline can be divided into 5 main steps: i.) Pre-processing of the histological images, ii.) Registration of the pre-processed ABA histology to the pre-processed experimental histology, iii.) tSNE of the SIMS dataset, iv.) Registration of the tSNE representation of the SIMS dataset to the experimental pre-processed histology, and v.) Final co-registration of the SIMS dataset and the ABA. A diagram of the complete co-registration workflow together with a more detailed description of all five steps are included in the Supplementary Information (Figure S1.) For more details the readers are kindly referred to the corresponding publications.

## Results

SIMS images tissue samples at the highest spatial resolution of current MSI techniques. Owing to the character of its probing beam, the spatial resolution of SIMS experiments can reach below 1 μm. Here we employed SIMS imaging to sagittal brain sections of *Mfp2* deficient mouse models. The MSI experiments were designed so that the cerebellum and brain stem were imaged at high spatial resolution. The pre-selection of the region of interest (ROI) was guided by pathological features such as cerebellar degeneration and neuroinflammation in the brainstem of *Mfp2*^*-/-*^ mice [21]. In addition, Oil Red O positive lipid deposits have been identified in the ependymal cells of the entire ventricular system and in cerebellar Bergmann glia fibers [21].

**Figure 1:**
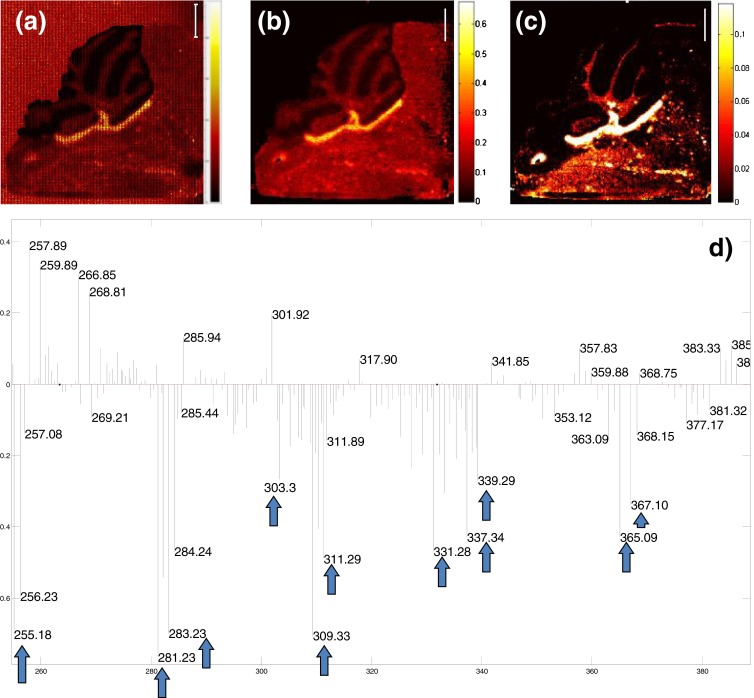
(**a**) Raw data MS image of *m/z* 281 (oleic acid 18:1), (**b**) MS image of *m/z* 281 after data pre-processing, (**c**) score image of pixels with negative score values of principal component (PC) number 3, (**d**) loadings plot corresponding to PC 3. The mass channels in the negative part of the loadings plot are associated with the hotspot highlighted on the score image (**c**). The mass channels marked with the blue arrows correspond to the following fatty acids (FA): *m/z* 255 (FA 16:0), *m/z* 281(FA 18:1), *m/z* 283 (FA 18:0), *m/z* 303 (FA 20:4), *m/z* 309 (FA 20:1), *m/z* 311 (FA 20:0), *m/z* 331 (FA 22:4), *m/z* 337 (FA 22:1), *m/z* 339 (FA 22:0), *m/z* 365 (FA 24:1), *m/z* 367 (FA 24:0). The peaks in the positive loading are speculated to originate from different lipid species, yet to be identified. (Shown is data from sample KO1. Validation results of the two biological replicates are included in Supplementary Information. See Figure S2.) Scale bar 1 mm

**Figure 2:**
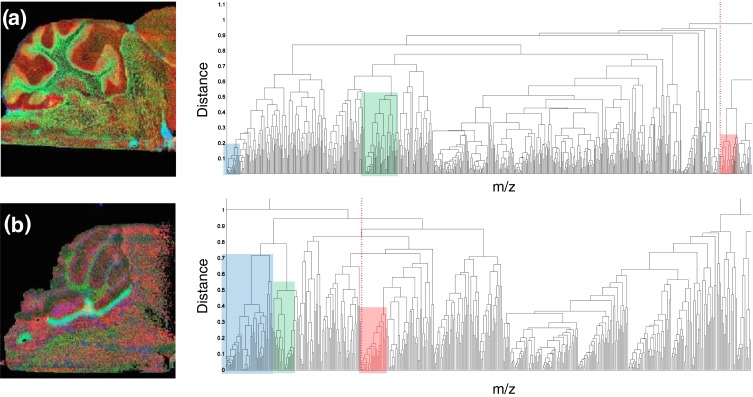
Comparison of hierarchical cluster analysis of (**a**) wild type and (**b**) knock-out mouse (KO1). Green and blue clusters represent fatty acids containing clusters. Red clusters contain chlorine. Although FA are present in the WT mouse, no accumulative pattern is observed

In the raw SIMS images of KO1 some lipid-related mass channels (e.g. oleic acid 18:1 at *m/z* 281) showed a significant spatial pattern manifested as a sharply denoted region beneath the cerebellum (Figure 1a). The pre-processing of the raw data significantly improved the image quality even though the spatial resolution was compromised due to the employed spatial binning (Figure 1b). PCA further highlighted the region which is clearly visible on the negative score image of the third principal component (PC) (Figure 1c). (*N.B.* The negative score image is an image of pixels with negative score values.) The negative part of the corresponding loadings plot shows, amongst others, mass channels belonging to various fatty acids (FA) – marked with blue arrows in Figure 1d. Most of them represent fatty acids with chain lengths ranging from 16 to 24 carbons. PCA enabled molecular characterization of the revealed hotspot through association of a specific molecular pattern. All three biological replicates manifested the presence of the fatty acids-related hotspot. The results of PCA for the two KO biological replicates are presented in the Supplementary Information (Figure S2.). The WT littermate of the MFP2 KO mouse models did not manifest the region-specific accumulation of fatty acids. Figure 2 shows results of HCA of WT and KO1 mouse. Shown are RGB overlays of three clusters: green and blue clusters contain mass channels of FA. Whereas a specific spatial pattern formed by the FA mass channels is formed in the KO model, no FA rich hotspot was observed in the WT mouse.

**Figure 3:**
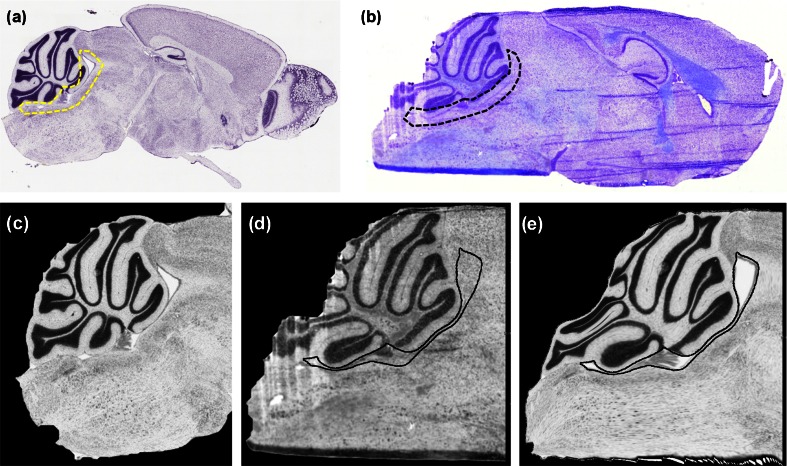
Comparison of the raw and pre-processed histologies. (**a**) The raw ABA reference section for the KO1, (**b**) the raw KO1 experimental section, (**c**) the pre-processed ABA histology, (**d**) the pre-processed experimental histology, and (**e**) the resulting image after registering the image in (**c**) to the image in (**d**). The black and yellow contour in panels (**a**) and (**b**) manually delineate the fourth ventricle and the cerebellar aqueduct. Contours in panels (**d**) and (**e**) represents the ABA VOIs of those particular structures

After SIMS imaging, the tissue sections were stained using Cresyl violet and optical images were recorded. Figure 3a and b show the ABA and the experimental histology for KO1, respectively. Based on their manual comparison and taking into account the shape of the observed FA hotspot, it was tentatively linked with the fourth ventricle and partly with the cerebellar aqueduct. However, in order to assess an unequivocal anatomic localization of the hotspot, the respective KO SIMS datasets were further coregistered to the corresponding ABA reference sections. Figure 3c and d show the pre-processed histologies of KO1 and their co-registration result, e. In black contour are shown the volumes of interest (VOIs), (i.e., corresponding ABA labels), of the fourth ventricle and the cerebellar aqueduct.

**Figure 4:**
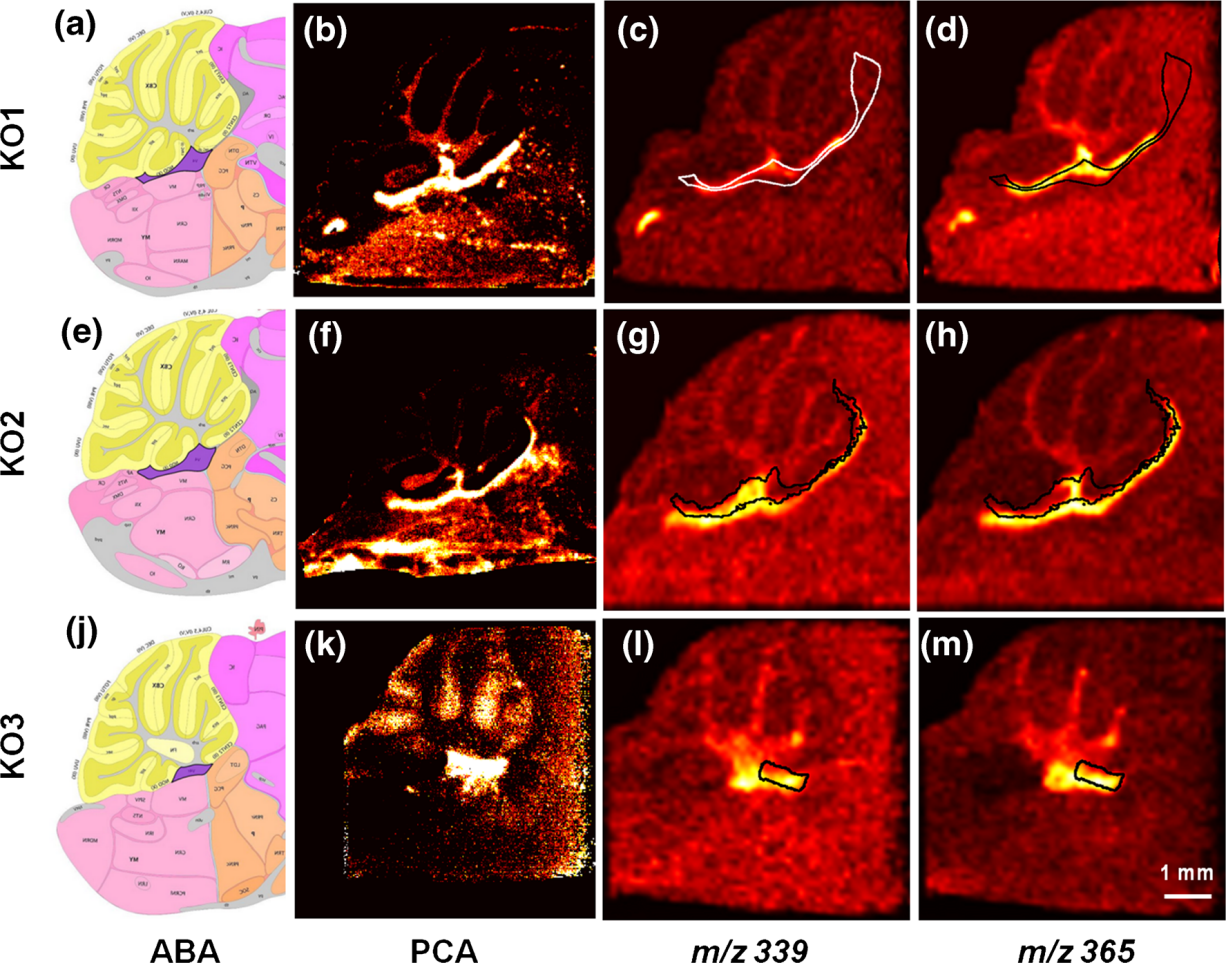
Results of the final ABA-MSI co-registration presented for three knock-out biological replicates (KO1, 2, 3). Panels (**a**), (**e**), and (**j**) show the anatomic segmentation map of the ABA reference section used for the co-registration pipeline of the respective samples. Highlighted in violet color is the region of the fourth ventricle. Panels (**b**), (**f**), and (**k**) show corresponding score images of the principal components that extracted the hotspots correlated with the presence of fatty acids in the three knock-outs. Panels (**c**), (**g**), (**l**), and (**d**), (**h**), (**m**), depict co-registration examples of two mass channels correlated with the hotspots (*m/z* 339 – fatty acid 22:0, and *m/z* 365 – fatty acid 24:1, respectively). In black (or white) is shown the label of the fourth ventricle (and for KO1 and KO2 also of cerebellar aqueduct). The scale bar indicates a 1 mm distance

Figure 4 shows the final MSI-ABA co-registration results for the three biological replicates. The analyzed tissue sections of the respective knock-outs were taken from different brain depths: the KO1 was cut approximately 700 μm deep away from the brain mid-line, the KO2 was sectioned right at the brain midline, and the KO3 section was acquired from a site more than 1 mm far from the mid-line. The cross-section of particular brain structures naturally changes within the brain depth. Also, the FA-rich region observed in all three KO replicates was manifested in different shapes. The ABA reference sections were selected as follows: KO1 – No.18, KO2 – No.21, and KO3 – No.16, based on their spacing in the ABA. The correct selection of the corresponding ABA reference section is essential as is further explained in the Discussion section. Figure 4a, e, and j show the anatomic segmentation maps with the VOIs of the selected ABA reference sections for the respective samples. The VOI of the fourth ventricle is highlighted in violet color. The shape of the fatty acids-related hotspot for the KO1–3 is in high contrast shown in Figure 4b, f, and k depicting the score images resulting from the PCA of the respective SIMS datasets. Figure 4c, g, l, and d, h, m show the final results of the SIMS-ABA co-registration. The VOI of the fourth ventricle (*V4r lateral recess*) is propagated onto the mass channels 339 (FA 22:0), and 365 (FA 24:1), respectively. The anatomic label is visible as black (or white where the contrast of the image requires) contour. Note that for KO1 and KO2 a VOI of the cerebellar aqueduct (*AQ*) was also projected onto the MS images. The label(s) and the hotspot co-localize, thus confirming the accumulation of the fatty acids in the fourth ventricle. Note that the VOIs extracted from the ABA and the FA hotspots demonstrate almost identical shapes, even though in some cases the hotspot signal does not fill the label to the full extent, hence making the link between the hotspot and the anatomic label stronger. The match is valid for all three KO replicates although the shape of the hotspot varies because of the different sectioning depths. Figure S3 in the Supplementary Information shows an overview of all FA mass channels labeled with the *V4r lateral recess* and *AQ* for KO1.

## Discussion

**Figure 5:**
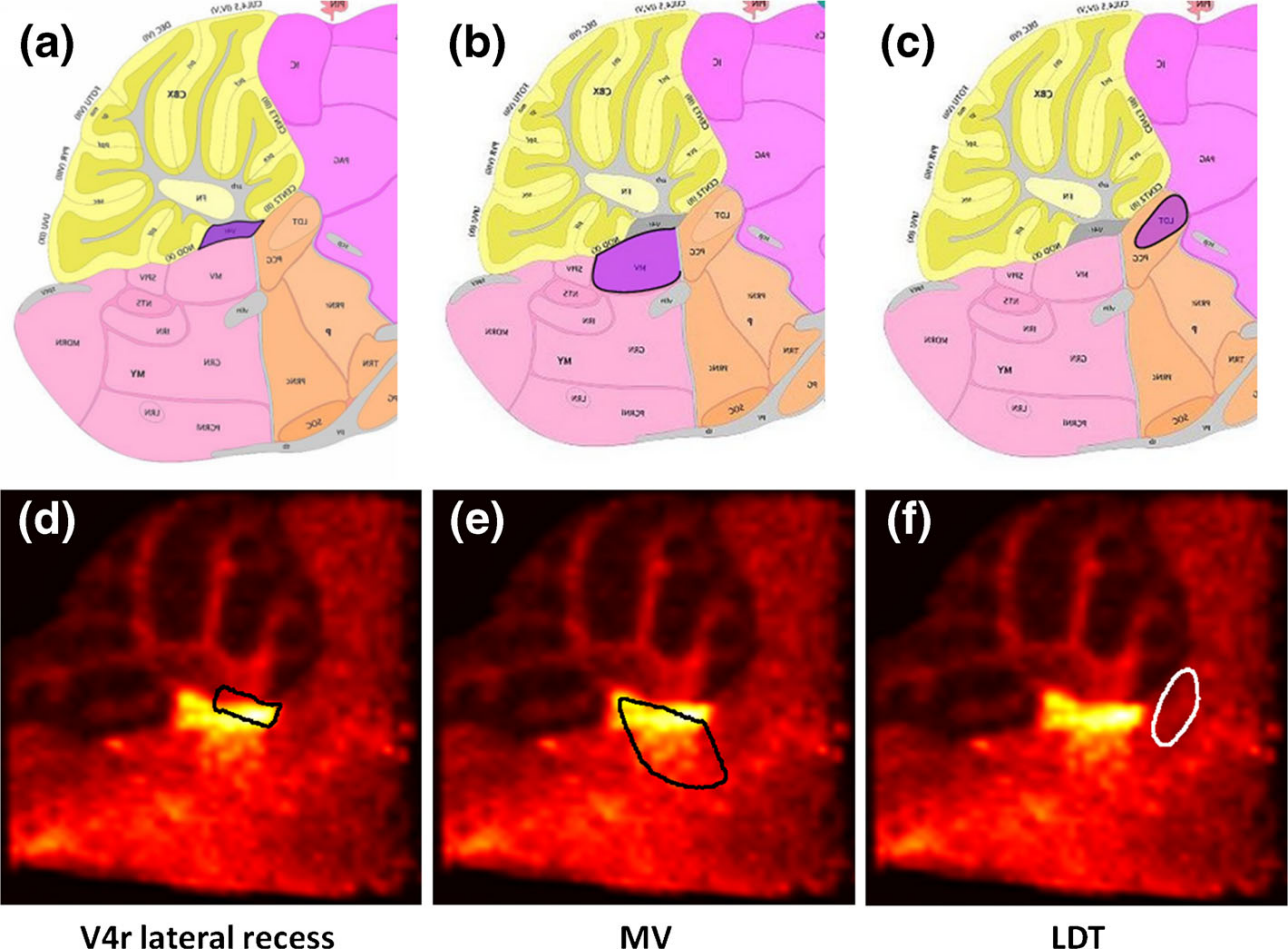
By comparing the anatomic segmentation map of the ABA reference section with the MSI data of the KO3, the hotspot can be assigned to several regions: most prominent candidates are (highlighted in violet color): (**a**) the fourth ventricle (*V4r lateral recess*), (**b**) the medial vestibular nucleus (*MV*), and (**c**) the laterodorsal tegmental nucleus (*LDT*). An accurate conclusion cannot be derived until the MSI-ABA co-registration is performed and the particular labels overlaid. Panels (**d**)–(**f**) show mass channel 309 (fatty acid 20:1) with the respective anatomic labels shown in black or white contour. The fourth ventricle shows by far the best match

MSI has the ability to extract anatomic features from tissue sections based on their molecular profiles. MSI distinguishes tissue regions even if they cannot be differentiated by conventional histochemical approaches. The combination of both, however, is beneficial as they provide complimentary information. Histochemical information, for instance, can be used to assign anatomic labels to the regions highlighted by MSI. Even though manual inspection is possible and represents an easy and fast way of MSI-anatomy comparison, it does not provide sufficient precision. Figure 5 shows the results of our ABA-MSI automated co-registration for KO3. A manual comparison of the ABA anatomic segmentation maps with the MSI data would assign different anatomic labels to the observed hotspot, namely, the fourth ventricle (*V4r lateral recess*) (Figure 5a), medial vestibular nucleus (*MV*) (Figure 5b), or laterodorsal tegmental nucleus (*LDT*) (Figure 5c) (*N.B.* The structures are highlighted in violet color in the respective anatomic maps.) The MSI-ABA co-registration allowed for correct anatomic identification. To demonstrate this, the listed labels were propagated onto the mass channel 339 (FA 22:0). From Figure 5d and e it is visible that the VOI *V4r lateral recess* matches the fatty acids hotspot. Even though the MSI signal does not fill in the whole VOI, the shapes of the hotspot, and the deformed *V4r lateral recess label* are close to identical.

Note that the quality of the co-registration process is highly dependent on the quality of the experimental tissue section and on the correct choice of the ABA reference section. Although the practice of MSI has improved tremendously over the past decade, there is still a fair amount of developmental work needed to refine the technique. The most important is the sample throughput. Its significant increase would allow for faster and maybe even automated methods development. To illustrate the time demands of a MSI experiment: the SIMS data acquisition of one of the described samples took roughly 15 h. Additional time is required for the sample preparation, data pre-processing and analysis, tissue staining, and the steps included in the MSI-ABA registration step. All combined, the amount of time required for a complete analysis of one sample is on the order of days.

SIMS is considered to be a “gentle” MS imaging method since it causes only superficial, minimal damage to a tissue surface. Even though it does not happen often, matrix-assisted laser desorption ionization (MALDI) MSI can also damage the tissue surface as the laser impact is much higher than the impact of the primary ion beam in SIMS. Some matrices, such as 9-aminoacridine, require high laser power. The laser ablated areas are often clearly visible on the tissue after histochemical staining. Tissue with such a changed morphology is especially challenging to retrospectively co-register, let alone to determine the identity of various anatomic features. Figure S4 in the Supplementary Information shows a comparison of a SIMS and a MALDI analyzed tissue section that was Nissl stained after the MSI experiments were completed. Nissl staining and, hence, the histology image quality of SIMS imaged samples can be hampered because of the gold layer often applied on top of the tissue surface. It causes a decrease in the dye intensity and makes the co-registration challenging. The staining procedure is further complicated by the use of the ITO glass slides required for the SIMS experiments. The thin layer of ITO compensates for the charge that builds up on top of the sample during the SIMS imaging. The tissue sections have the tendency to float away from the glass surface during the staining. If not performed with special care, the tissue can be damaged during the staining protocol. (Note that the SIMS imaged tissue section of KO2 was damaged during the staining process. The MALDI imaged adjacent tissue section – shown in Figure S4b, Supplementary Information) – was thus used in the KO2 co-registration pipeline.)

The choice of the correct ABA reference section is crucial to the success of the co-registration procedure. Whereas the ABA features 132 coronal mouse brain sections evenly spaced at 100 μm, there are only 21 sections in the sagittal plain. The sagittal sections were taken only from one hemisphere as the left and right hemispheres are anatomically symmetrical. The narrower width of the brain in sagittal plain also contributes to the lower number of the sections. Nevertheless, the sagittal sections in the ABA were taken at 200 μm spacing. In practice, it means that 16 twelve-micron-thick experimental tissue sections fit to only one ABA reference section. On one hand, the choice of the correct reference section is less difficult, on the other hand, it is less accurate, and thus can cause imprecision in the histology-to-histology registration step.

Accurate and reproducible sample preparation should always be at the top of the researcher’s priority list. Recently, a paper devoted to a good practice for tissue sectioning and general sample preparation before MALDI MSI was published and features several tips and tricks [22]. Sectioning with anatomic atlas as a reference is recommended, especially for complex tissue samples such as brain.

Our approach assisted in the assignment of the correct anatomic label to the hotspot of FA accumulated in the *Mfp2*^*-/-*^ mouse models. MFP2 is a protein that catalyzes specific reactions of the peroxisomal β-oxidation. This process is responsible for chain shortening of several carboxylate substrates, among others the VLCFA. Patients with severe MFP2 deficiency suffer from neonatal hypotonia, brain malformations, seizures, and psychomotor retardation, and their life time typically does not exceed 1 y. [23]. Similarly, the KO mouse models manifest motor impairment, ataxia, and die within 6 mo. [23]. Oil Red O staining of the brain sections of *Mfp2*^*-/-*^ mice showed significant lipid accumulation within several brain regions, most specifically within the ependymal cells of the whole ventricular system [21]. Dinkel et al. [24] revealed that *Mfp2* is expressed in the ependymal cells isolated from the fourth ventricle. Ependymal cells play a role in production of the cerebrospinal fluid (CSF).

We discovered that in MFP2 knock-outs, FAs accumulate in the fourth ventricle and in the cerebellar aqueduct. As the latter connects the fourth and the third ventricle, other parts of the brain will also be exposed to the lipids. Moreover, Borges et al. demonstrated in 1985 that Purkinje cells can selectively accumulate certain low- and high-molecular mass compounds from the CSF [25]. Purkinje cells provide the sole output of the cerebellum and project to the vestibular and deep cerebellar nuclei in order to control body movement and coordination. These finding suggest that in the *Mfp2*^*-/-*^ mice, the function of Purkinje cells or the related structures such as the cerebellar nuclei can be influenced by the VLCFA accumulation in the fourth ventricle.

Because of the high impact energy of the primary ion beam in SIMS, we suggest that the observed VLCFA result from fragmentation of the intact lipids. Whether these are glycerophospholipids, sphingolipids, triglycerides, or cholesterylesters could not be determined. Whereas very long chain fatty acids with chain lengths of 22 or more carbons are selective substrates for peroxisomal β-oxidation, the shorter ones can be degraded by mitochondria, and it is not clear why they accumulate in MFP2-deficient brain. As the MSI methodology does not allow quantification, it would be interesting to determine the relative contribution of each of the fatty acids by sampling CSF from the mice.

The ABA further features an application that allows for comparison of genes with similar expression pattern in terms of neuroanatomic distribution. The comparison is based on Pearson’s correlation. The function produces a list of genes sorted according to their decreased correlation with the targeted gene (i.e., according to decreasing values of the Pearson’s coefficient). Da Silva et al. [26] published a gene correlation study based on the ABA tool. Their targeted gene was SLC20A2 whose mutations cause the familial idiopathic basal ganglia calcification (Fahr’s disease) molecularly demonstrated as deposits of calcium in basal ganglia and other distinct brain regions. The group took the advantage of The Brain Explorer (a tool offered by the ABA) to quantitatively assess expression of the SLC20A2 gene in different brain regions. MFP2 is encoded by the 17β-hydroxysteroid dehydrogenase 4 gene (*Hsd17b4*) [17]. We applied the ABA search tool to find genes correlated with *Hsd17b4*. The search listed the total number of 977 genes with various degrees of (anti-)correlation. Da Silva et al. used as a cut-off for the genes of interest r ≥ 0.5 for the correlates, and r ≤ –0.5 for the anti-correlates. We summarized the first 10 hits of the correlates in Table S1, which you can find in the Supplementary Information. The search found only one gene with Pearson’s coefficient lower than –0.5. The listed genes were examined through the UniProt [26] database and explanation of their function (if available) was provided. The fatty aldehyde dehydrogenase (*Aldh3a2*) showed the highest correlation of all genes. It catalyzes the oxidation of long-chain aliphatic aldehydes to fatty acids, and is responsible for conversion of the sphingosine 1-phosphate degradation product hexadecenal to hexadecenoic acid [26]. It is also considered to be involved in the degradation of phytanic acid, a saturated branched chain fatty acid [27, 28]. Another possibility is to search for genes that are expressed in particular brain regions. In our example, the cells adjacent to the fourth ventricle (i.e., ependymal cells and choroid plexus) were unfortunately not included in the structural list. Further deep exploration of the genes related to MFP2 and the associated anatomic structures might provide more information on the molecular mechanism of persoxisomal dysfunctions.

## Conclusions

The presented work demonstrates the potential of the automated co-registration of mass spectrometry imaging (MSI) data to curated anatomic atlases for precise anatomic localization of the MSI-derived molecular signatures. We employed a fully automated pipeline to co-register brain MSI data of *Mfp2* deficient mouse models to the Allen Brain Atlas (ABA). High resolution secondary ion mass spectrometry imaging of the knock-out murine brains revealed accumulation very long chained fatty acids in specific brain regions. The advantage of high spatial resolution MSI is the enhanced similarity to histological images which improves automated co-registration. Manual comparison of SIMS images with the histological information provided by the Nissl stained tissue sections did not allow for an unequivocal annotation of the FA rich brain regions. The result of MSI-ABA co-registration linked the FA hotspot to the 4^th^ ventricle. The precise localization of the MSI signal brought new insights into the molecular mechanism of *Mfp2* deficiency. The ABA tools were further used to generate a list of genes correlated with the MFP2 expression pattern. Various molecular substrates linked to the correlated genes or to the structures linked with the 4th ventricle can be used for future targeted molecular analysis of *Mfp2*^*-/-*^ mouse models.

## Electronic supplementary material

ESM 1 (DOCX 9585 kb)
