# Supplementary material for: Precise Anatomic Localization of Accumulated Lipids in Mfp2 Deficient Murine Brains Through Automated Registration of SIMS Images to the Allen Brain Atlas
Source: J Am Soc Mass Spectrom. 2015 Apr 28;26(6):948–57. doi: 10.1007/s13361-015-1146-6 (PMC4422856; doi:10.1007/s13361-015-1146-6)
Supplement: Supplementary file 1 — (DOCX 9585 kb) [file 13361_2015_1146_MOESM1_ESM.docx]

**SUPPLEMENTARY INFORMATION**

**Precise anatomical localization of accumulated lipids in *Mfp2* deficient murine brains through automated registration of SIMS images to the Allen Brain Atlas**

Karolina Škrášková^1,2^; Artem Khmelinskii^1,3,4^; Walid M. Abdelmoula^4^; Stephanie De Munter^5^; Myriam Baes^5^; Liam McDonnell^6,7^; Jouke Dijkstra^4^; and Ron M.A. Heeren^1,2,8^

^1^ FOM Institute AMOLF, Amsterdam The Netherlands

^2^ TI-COAST, Amsterdam, The Netherlands

^3^ Percuros B.V., Enschede, The Netherlands

^4^ Division of Image Processing, Department of Radiology, LUMC, Leiden, The Netherlands

^5^ Laboratory of Cellular Metabolism, KU Leuven, Leuven, Belgium

^6^ Center for Proteomics and Metabolomics Leiden University Medical Center, 2333 ZA

Leiden, The Netherlands

^7^ Fondazione Pisana per la Scienza ONLUS, 56121 Pisa, Italy

^8^ M4I, The Maastricht MultiModal Molecular Imaging Institute, University of Maastricht, Maastricht, The Netherlands

**Corresponding author**

Prof. Dr. Ron M.A. Heeren

[r.heeren@maastrichtuniversity.nl](mailto:r.heeren@maastrichtuniversity.nl)

M4I, The Maastricht MultiModal Molecular Imaging Institute, University of Maastricht, Universiteitssingel 50, 6229 XR Maastricht, The Netherlands

**Table of content**

**Page 2:** Figure S1. Diagram and description of the complete co-registration workflow.

**Page 4**: Figure S2. Validation of PCA results for two KO biological replicates.

**Page 5**: Figure S3. Overview off all fatty acids mass channels for KO1 co-registered to the ABA.

**Page 6:** Figure S4. Comparison of SIMS and MALDI imaged and subsequently Nissl stained. tissue.

**Page 7:** Table 1. List of genes with a similar expression pattern as manifests the gene *Hsd17b4.*


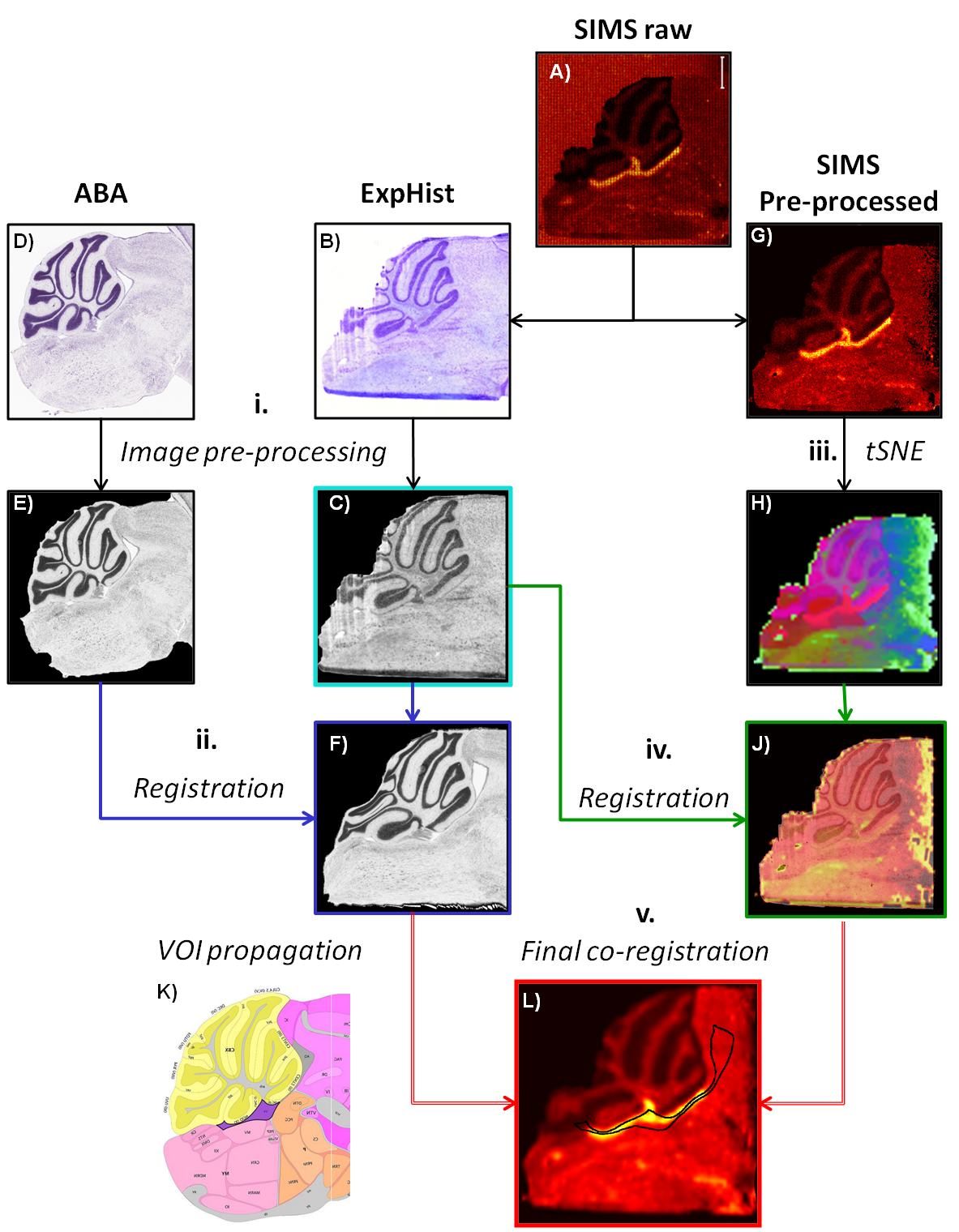


**Figure S1. Diagram and description of the complete co-registration workflow.** The pre-processed high resolution experimental histology (panel C) plays a pivotal role in the co-registration pipeline. It serves as an intermediate between mass spectrometry imaging (MSI) data and the Allen Brain Atlas (ABA). The whole co-registration pipeline can be divided into 5 main steps:

*i.) Pre-processing of the histological images*

Both the experimental histology (Figure S1.B) and the ABA reference section from the experiment-matching brain depth (Figure S1.D) were pre-processed to exclude background noise, correct for potential image acquisition artifacts, and to maximize the contrast. The images were classified into two clusters using k-means clustering, followed by morphological operations to close any potential gaps in the clustered images. The resulting binary masks were used to separate the tissue from the background.

*ii.) Registration of the pre-processed ABA histology (Figure S1.E) to the pre-processed experimental histology* *(Figure S1.C)*

Registration was performed in a coarse-to-fine process. First, an affine registration was performed for rough global alignment and scaling. Subsequently, a multi-resolution non-rigid B-spline registration was applied. A Gaussian image pyramid was employed in all registration steps applying three resolutions for the affine, and twelve for the B-spline part. Mutual information was used as a similarity metric. The registration was implemented using the open source image registration toolbox Elastix [1]. For more details see reference [2].

*iii.) tSNE of the SIMS dataset*

tSNE was applied on the pre-processed SIMS dataset (Figure S1.G) to find its low-dimensionality representation. It was performed using the default settings described in [3] and the tSNE Matlab toolbox [4].

*iv.) Registration of the tSNE representation of the SIMS dataset (Figure S1.H) to the experimental pre-processed histology (Figure S1.C)*

The tSNE image was registered to the experimental histological image and the obtained deformation field (Figure S1.J) was used to deform each mass channel to the histological reference space. For more details see reference [5].

*v.) Final co-registration of the SIMS dataset and the ABA*

Using the information provided by the deformation fields obtained in steps ii.) and iv) the selected ABA anatomical labels (volumes of interest – VOIs, Figure S1.K), were finally propagated onto each mass channel of interest (Figure S1.L). The VOIs are hierarchically organized, and sorted through the ABA anatomical segmentation maps.

**Figure S2. Validation of principal component analysis results the for two knock-out biological replicates.** Shown are score images of principal components that highlighted the tissue region localized underneath the cerebellum, KO2 in panel **a)**, and KO3 in panel **c)**. The ranking of the principal components (PCs) showing the hotspot differed. Whereas for KO1 and KO2 the hotspot was visible on PCs 3, KO3 highlighted the hotspot through PC 6. Panels **b)**, and **d)** show the corresponding loadings plots with the fatty acids marked via blue arrows.

**Figure S3. Overview off all fatty acids mass channels for KO1 co-registered to the ABA.** Shown are FA mass channels which were correlated with the hotspot via the principal component analysis. Highlighted in black is the anatomical region of the 4th ventricle and the cerebellar aqueduct. The mass channels correspond to the following fatty acids: *m/z* 255 FA 16:0, *m/z* 281FA 18:1, *m/z* 283 FA 18:0, *m/z* 303 FA 20:4, *m/z* 309 FA 20:1, *m/z* 311 FA 20:0, *m/z* 331 FA 22:4, *m/z* 337 FA 22:1, *m/z* 339 FA 22:0, *m/z* 365 FA 24:1, and *m/z* 367 FA 24:0.

**Figure S4. Comparison of SIMS and MALDI imaged and subsequently Nissl stained. tissue.**

Whereas SIMS is considered as a non-invasive ionization technique that causes minimal damage / changes to the sample surface, the impact of laser during a MALDI experiment can sometimes significantly change the tissue morphology. **a)** SIMS imaged Nissl stained experimental tissue section for KO1 without surface changes. **b)** MALDI imaged Nissl stained tissue section with clearly visible imprints of the laser shots. Drawing anatomical conclusion from such a damaged tissue section is difficult if not impossible. Note that the SIMS imaged tissue section of KO2 was damaged during the staining process. The MALDI imaged adjacent tissue section was thus used in the KO2 co-registration pipeline.

| **Gene abbrev** | **Gene name** | **Pearson Coefficient** | **Notes** |
| --- | --- | --- | --- |
| Aldh3a2 | Aldehyde dehydrogenase family 3, subfamily A2 | 0.864 | Catalyzes the oxidation of long-chain aliphatic aldehydes to fatty acids. Responsible for conversion of the sphingosine 1-phosphate (S1P) degradation product hexadecenal to hexadecenoic acid |
| Atg13 | Autophagy related 13 | 0.838 |  |
| Brox | BRO1 domain and CAAX motiff containing | 0.832 | Subcellular location: Lipid anchor = Protein bound to the lipid bilayer of a membrane through a posttranslationally modification by the attachment of at least one lipid or fatty acid, e.g. farnesyl, palmitate and myristate. |
| Mphosph6 | M-phase phosphoprotein 6 | 0.814 |  |
| Pddc1 | Parkinson disease 7 domain-containing protein 1 | 0.777 |  |
| Wdr5 | WD repeat domain 5 | 0.770 |  |
| Tacc2 | Transforming acidic coiled-coil-containing protein 2 | 0.757 |  |
| Fam133b | Protein FAM133B | 0.751 |  |
| Mrps16 | 28S ribosomal protein S16, mitochondrial | 0.741 |  |
| Hnrnpk | Heterogeneous nuclear ribonucleoprotein K | 0.732 |  |
| Cacna1a | Voltage-dependent P/Q-type calcium channel subunit alpha-1A | -0.879 | Voltage-sensitive calcium channels (VSCC) mediate the entry of calcium ions into excitable cells and are also involved in a variety of calcium-dependent processes, including muscle contraction, hormone or neurotransmitter release, gene expression, cell motility, cell division and cell death. |

**Table 1. List of genes with a similar expression pattern** (in the terms of neuroanatomical distribution) as manifests the gene *Hsd17b4* (the gene encoding the MFP2 protein). The correlation calculation is based on Pearson’s coefficient. The calculation was done via tool provided by the ABA. Listed are 10 most correlated genes. *Cacna1 a* showed on the contrary high anti-correlation (the only gene with PC lower that -0.5).

(1) Klein, S., Staring, M., Murphy, K., Viergever, M. A., Pluim, J. P., Elastix: A toolbox for intensity-based medical image registration. *IEEE Trans Med Imaging* **2010**, *29,* 196-205.

(2) Abdelmoula, W. M., Carreira, R. J., Shyti, R., Balluff, B., van Zeijl, R. J., Tolner, E. A., Lelieveldt, B. F., van den Maagdenberg, A. M., McDonnell, L. A., Dijkstra, J., Automatic registration of mass spectrometry imaging data sets to the allen brain atlas. *Anal Chem* **2014**, *86,* 3947-3954.

(3) van der Maaten, L., Hinton, G., Visualizing data using t-sne. *The Journal of Machine Learning Research* **2008**, *9,* 85.

(4) <http://homepage.tudelft.nl/19j49/t-SNE.html>.

(5) Abdelmoula, W. M., Skraskova, K., Balluff, B., Carreira, R. J., Tolner, E. A., Lelieveldt, B. F., van der Maaten, L., Morreau, H., van den Maagdenberg, A. M., Heeren, R. M., McDonnell, L. A., Dijkstra, J., Automatic generic registration of mass spectrometry imaging data to histology using nonlinear stochastic embedding. *Anal Chem* **2014**.
